# Supplementary material for: A GPU-based computational framework that bridges neuron simulation and artificial intelligence
Source: Nat Commun. 2023 Sep 18;14:5798. doi: 10.1038/s41467-023-41553-7 (PMC10507119; doi:10.1038/s41467-023-41553-7)
Supplement: Supplementary file 1 — Supplementary Information [file 41467_2023_41553_MOESM1_ESM.pdf]

Supplementary Information

A GPU-based computational framework that bridges Neuron simulation and Artificial Intelligence

Yichen Zhang[1#], Gan He[1#], Lei Ma[1,2#], Xiaofei Liu[1,3], J.J.Johannes Hjorth[4], Alexander Kozlov[4,5], Yutao He[1], Shenjian Zhang[1], Jeanette Hellgren Kotaleski[4,5], Yonghong Tian[1,6], Sten Grillner[5], Kai Du[7*] & Tiejun Huang[1,2,7]

[1] National Key Laboratory for Multimedia Information Processing, School of Computer Science, Peking University, Beijing 100871, China.

[2] Beijing Academy of Artificial Intelligence (BAAI), Beijing 100084, China.

[3] School of Information Science and Engineering, Yunnan University, Kunming 650500, China.

[4] Science for Life Laboratory, School of Electrical Engineering and Computer Science, Royal Institute of Technology KTH, Stockholm SE-10044, Sweden.

[5] Department of Neuroscience, Karolinska Institute, Stockholm SE-17165, Sweden.

[6] School of Electrical and Computer Engineering, Shenzhen Graduate School, Peking University, Shenzhen 518055, China.

[7] Institute for Artificial Intelligence, Peking University, Beijing 100871, China.

[#] These authors contributed equally: Yichen Zhang, Gan He, Lei Ma.

* Correspondence: kai.du@pku.edu.cn

Ours	Phase	Compared method	Model	Speedup
DHS	solving equations	CoreNEURON method (GPU)	pyramidal	10.08-11.35
			Purkinje	13.11-17.00
			CA3b	11.76-15.00
			CA1	11.78-13.63
			mitral	7.85-9.14
			SPN	9.39-12.56
		serial Hines method (CPU)	pyramidal	174.11-953.65
			Purkinje	285.67-1589.42
			CA3b	145.43-800.48
			CA1	238.41-1162.62
			mitral	127.85-713.91
			SPN	67.10-270.73
DeepDendrite	simulation	serial NEURON (CPU)	full-spine model	96.37
		CoreNEURON (GPU)	full-spine model	8.19
		serial NEURON (CPU)	few-spine model	131.42
		CoreNEURON (GPU)	few-spine model	6.71
	training	40-process-parallel NEURON (CPU)	HPC-Net	25.37
	testing	40-process-parallel NEURON (CPU)	HPC-Net	26.54

Supplementary Table 1 | Summary of speedup referred to in our experiments. CoreNEURON version: 0.14, NEURON version: 7.6.2; CPU: Intel Xeon E5-2698 v4, GPU: NVIDIA Tesla A100.

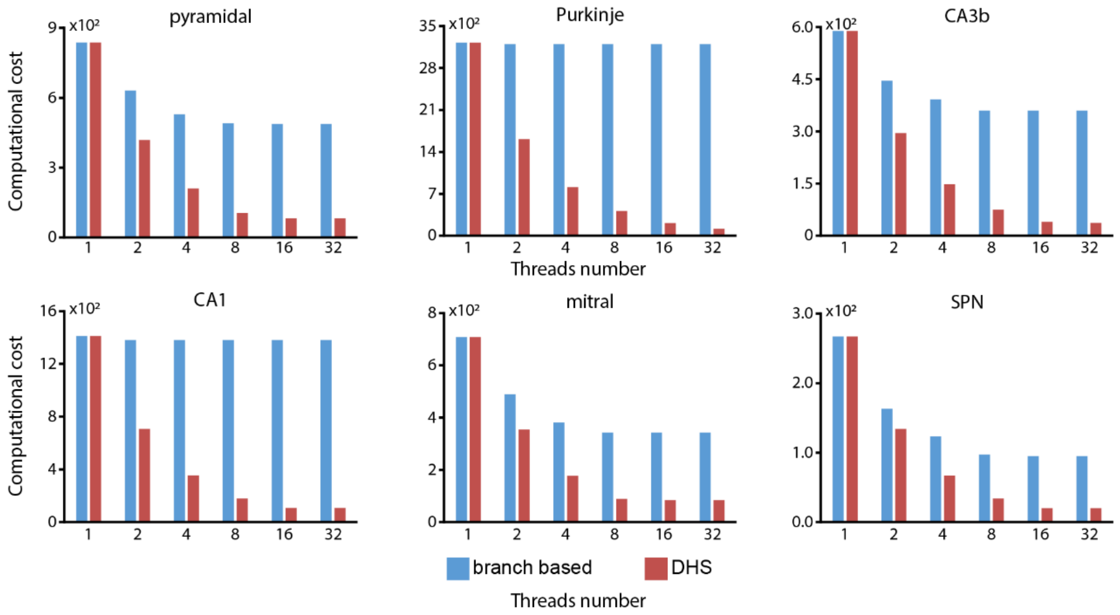

Supplementary Figure 1 | DHS outperforms the branch-based method in computational cost. DHS achieves low computational cost on various types of neurons while the cost of the branch-based method is much higher. The cost of the branch-based method remains high despite the growth of thread number, and in some cases (e.g., Purkinje cell and CA1 cell) only little gain is achieved.

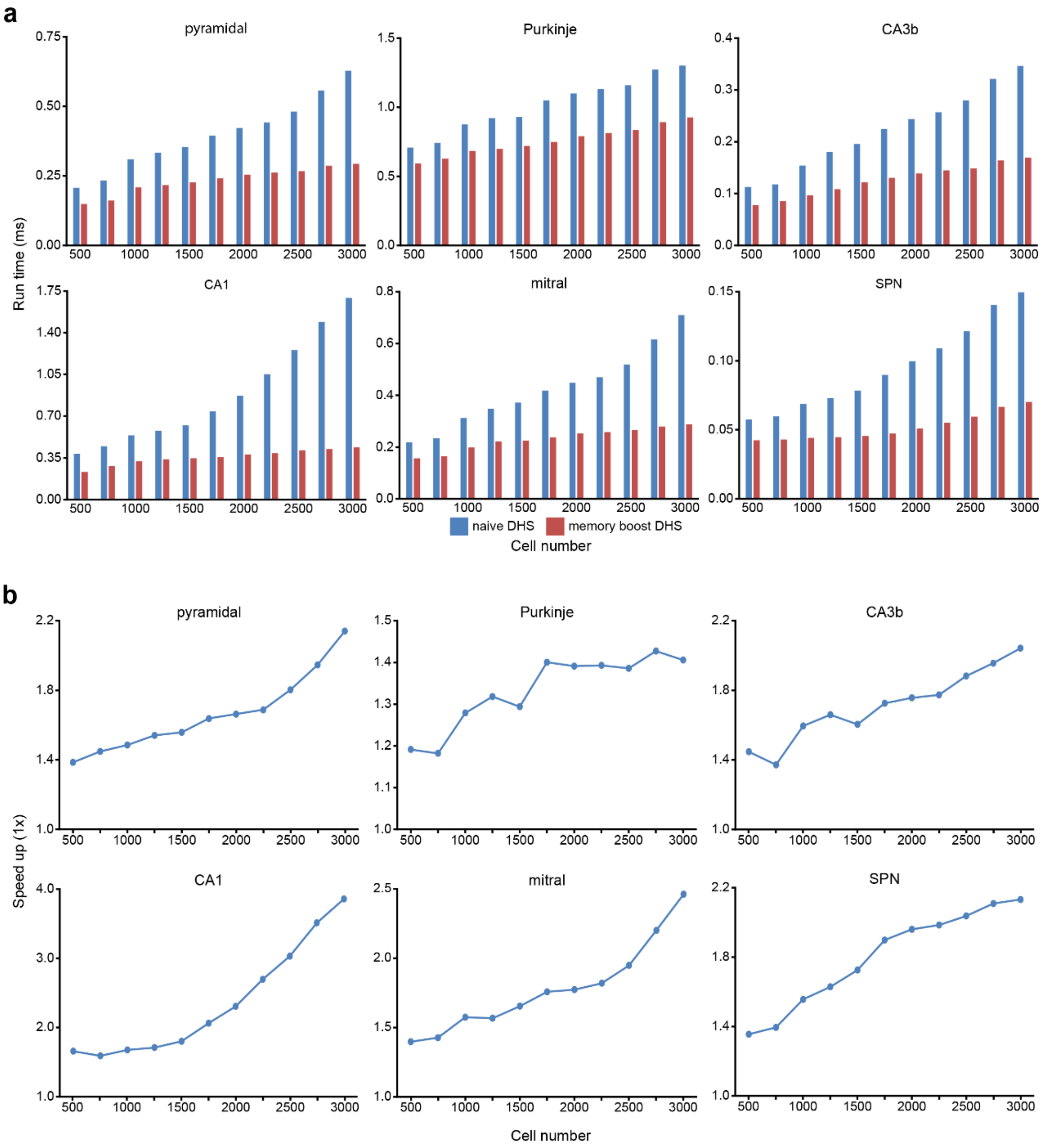

Supplementary Figure 2 | GPU memory boosting further speeds up DHS. a GPU memory boosting reduces the run time of DHS on various types of neurons. **b** With GPU memory boosting, DHS becomes 1.2-3.8 times faster on different types of neurons

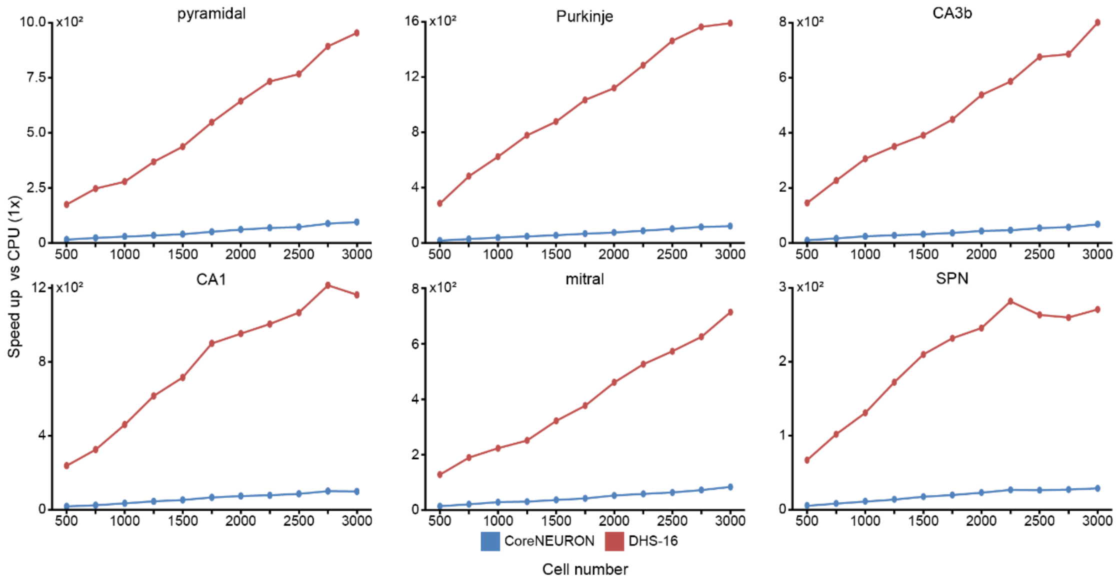

Supplementary Figure 3 | DHS achieves 2-3 orders of magnitude speedup compared to the serial Hines method on CPUs. On the test neurons used in this study, the GPU parallel method used in CoreNEURON achieves a speedup of 2.5-120 times compared to the serial Hines method on CPU. DHS method is 7-17 times faster than the method in CoreNEURON and achieves a speedup of 60-1,500 times compared to the serial Hines method on CPU.

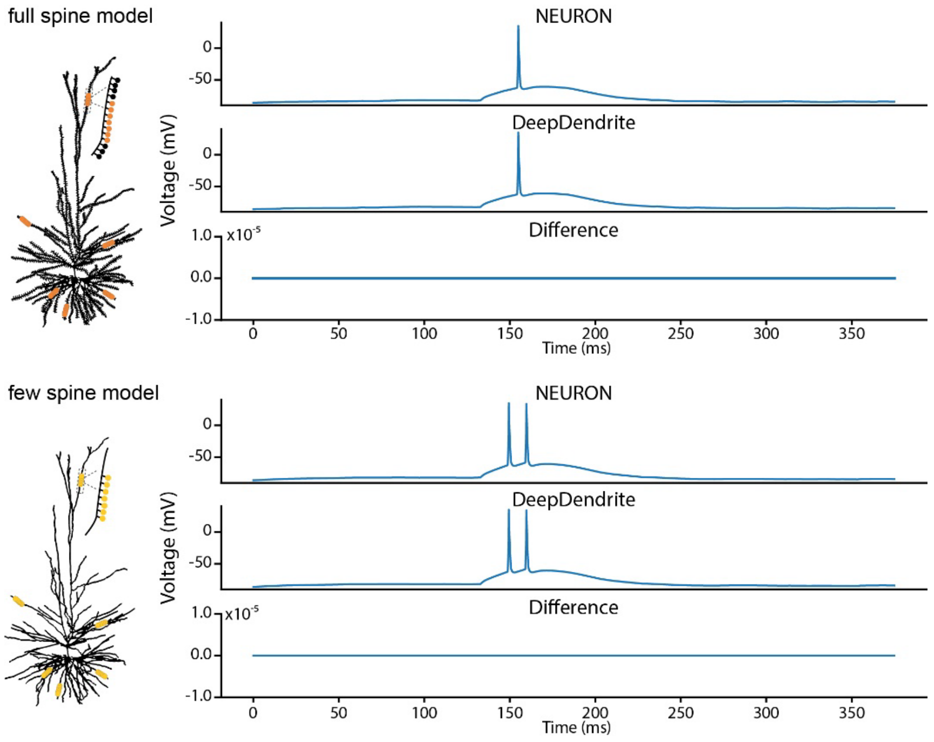

Supplementary Figure 4 | DeepDendrite can get identical numerical accuracy as NEURON. The simulation results of the full-spine model (top) and the few-spine model (bottom) in Fig. 5 are shown here. The recorded somatic voltages from NEURON and DeepDendrite are identical.

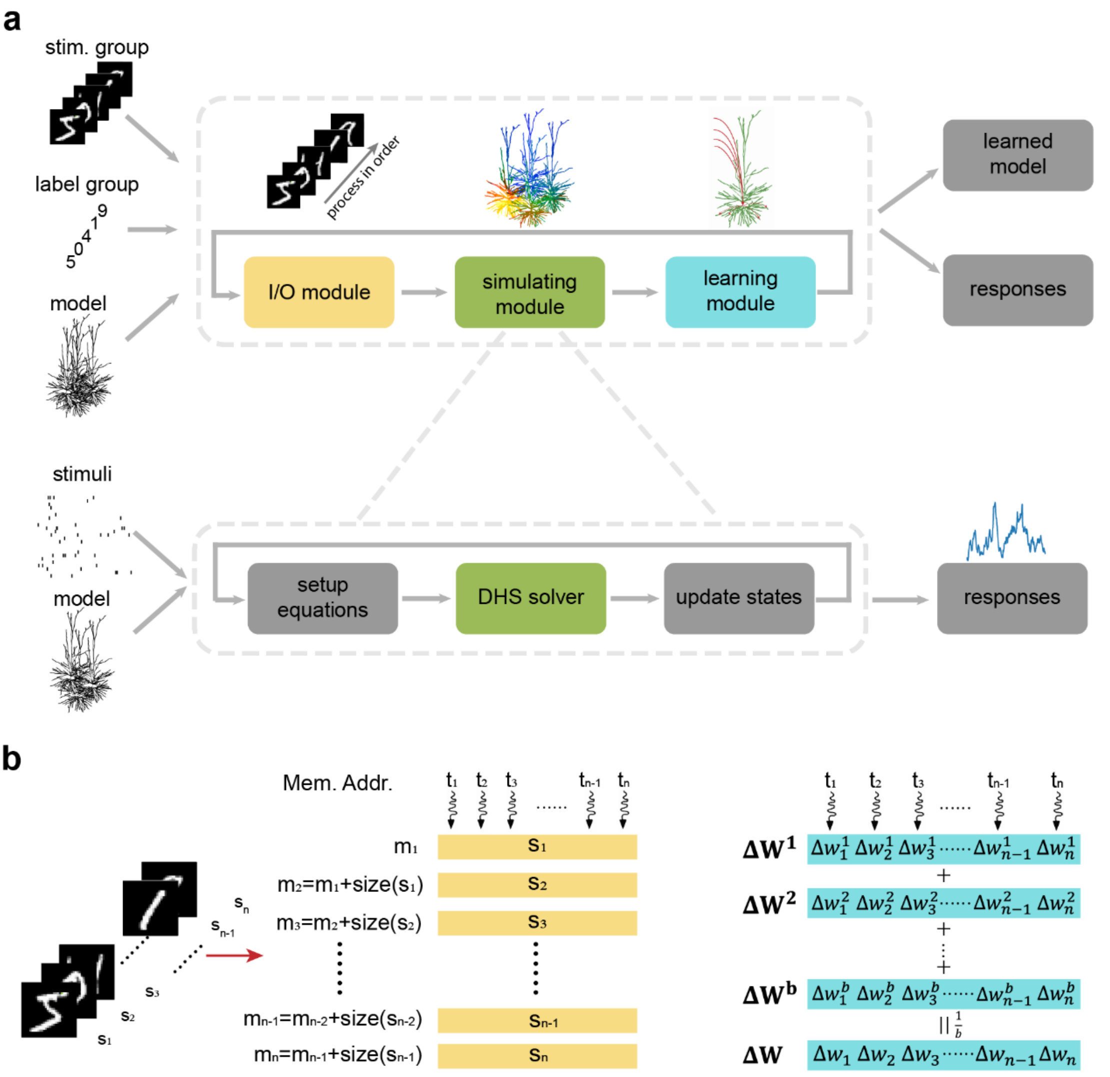

c

Algorithm 1 Training on DeepDendrite

Input Model M, Training Set $TrainSet$, Training Set size N, batch size b, iteration number N_{iter}
Output Weights after training $\mathbf{W}$

function GPUTRAIN(M, $TrainSet, N, b, N_{iter}$)
  $istim = 0$
  $iter = 0$
  while $iter < N_{iter}$ **do**
    $\mathbf{s}, \mathbf{l} = TrainSet[istim : (istim + b)\%N]$
    Attach stimuli $\mathbf{s}$ and labels $\mathbf{l}$ to the model M
    Simulate to get the responses of all neurons
    Compute $\Delta\mathbf{W}_1, \ldots \Delta\mathbf{W}_b$
    Set $\Delta\mathbf{W} = mean(\Delta\mathbf{W}_1, \ldots \Delta\mathbf{W}_b)$
    Update all synaptic weights $\mathbf{W} = \mathbf{W} + \eta\Delta\mathbf{W}$
    $istim = (istim + b)\%N$
    $iter += 1$
  end while
end function

Algorithm 2 Testing on DeepDendrite

Input Model M, Test Set $TestSet$, Set size N, batch size b
Output Responses of the model $\mathbf{Y}_{pred}$

function GPUTEST(M, $TestSet, N, b$)
  $istim = 0$
  while $istim < N$ **do**
    $\mathbf{s}, \mathbf{l} = TestSet[istim : istim + b]$
    Attach stimuli $\mathbf{s}$ to the model
    Simulate to get all neural responses $y_1, y_2 \ldots y_b$
    Set $\mathbf{Y}_{pred}[istim : istim + b] = \{y_1, y_2, \ldots, y_b\}$
    $istim = (istim + b)\%N$
  end while
end function

Supplementary Figure 5 | The framework and implementation of DeepDendrite. a The DeepDendrite framework**.** DeepDendrite consists of three modules: an I/O module, a simulating module, and a learning module. During learning, DeepDendrite takes the

detailed model and all training samples as input and saves the model after learning. During each iteration in training, the I/O module picks specific stimuli from all training samples and attaches them to the detailed model. Then simulating module starts the simulation and gets the responses of the detailed model. After the simulation, the learning module updates the synaptic weights according to the network responses and the target signal. **b** GPU implementation and optimization of the I/O module (left) and the learning module (right). **c** The procedure of training (left) and testing (right) when performing AI tasks with detailed network models on DeepDendrite.

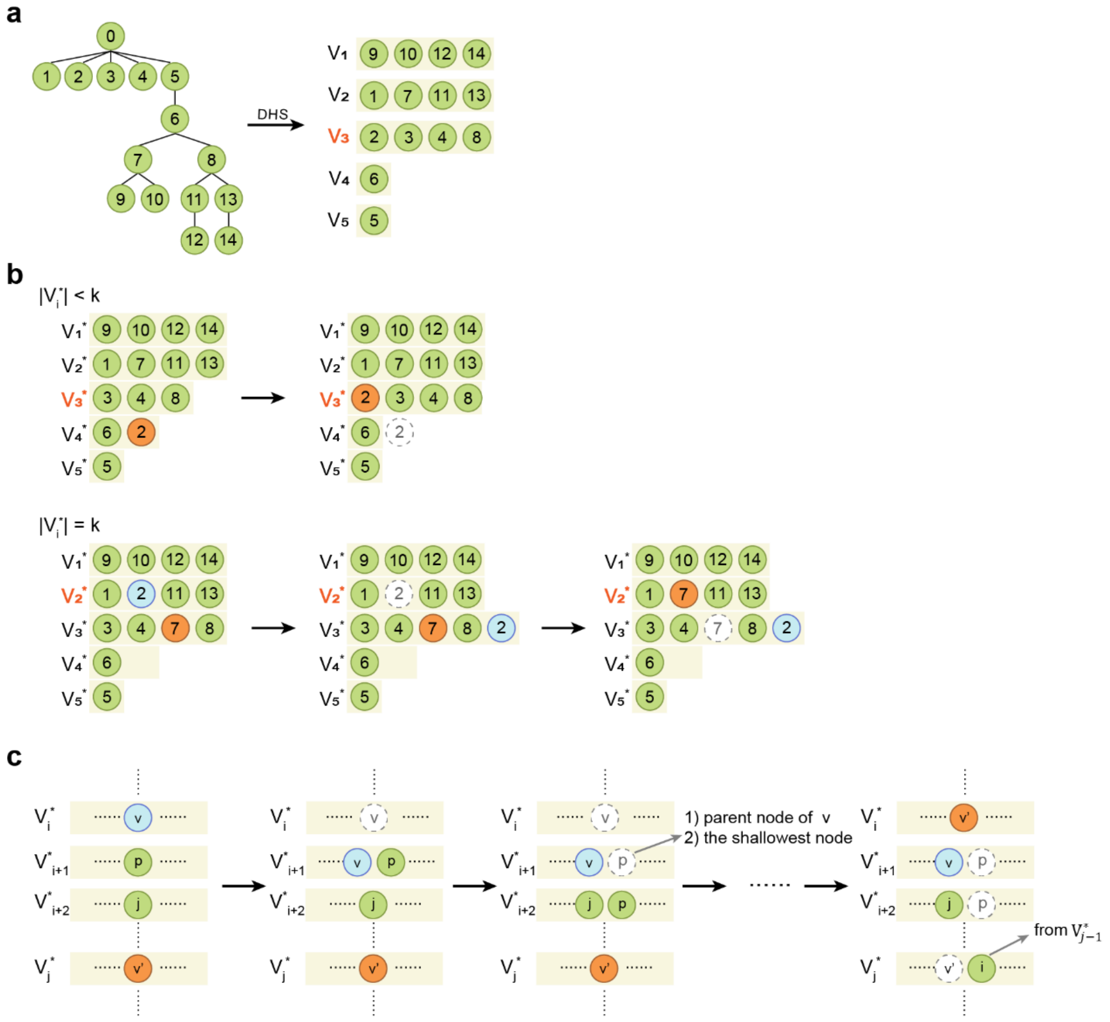

Supplementary Figure 6 | Strategy for modifying subset V_i^* to make it satisfy the max-depth criteria. a Partition generated by DHS. **b** A simple example showing how to modify subset V_i^*. **c** The general case when $|V_i^*| = k$.

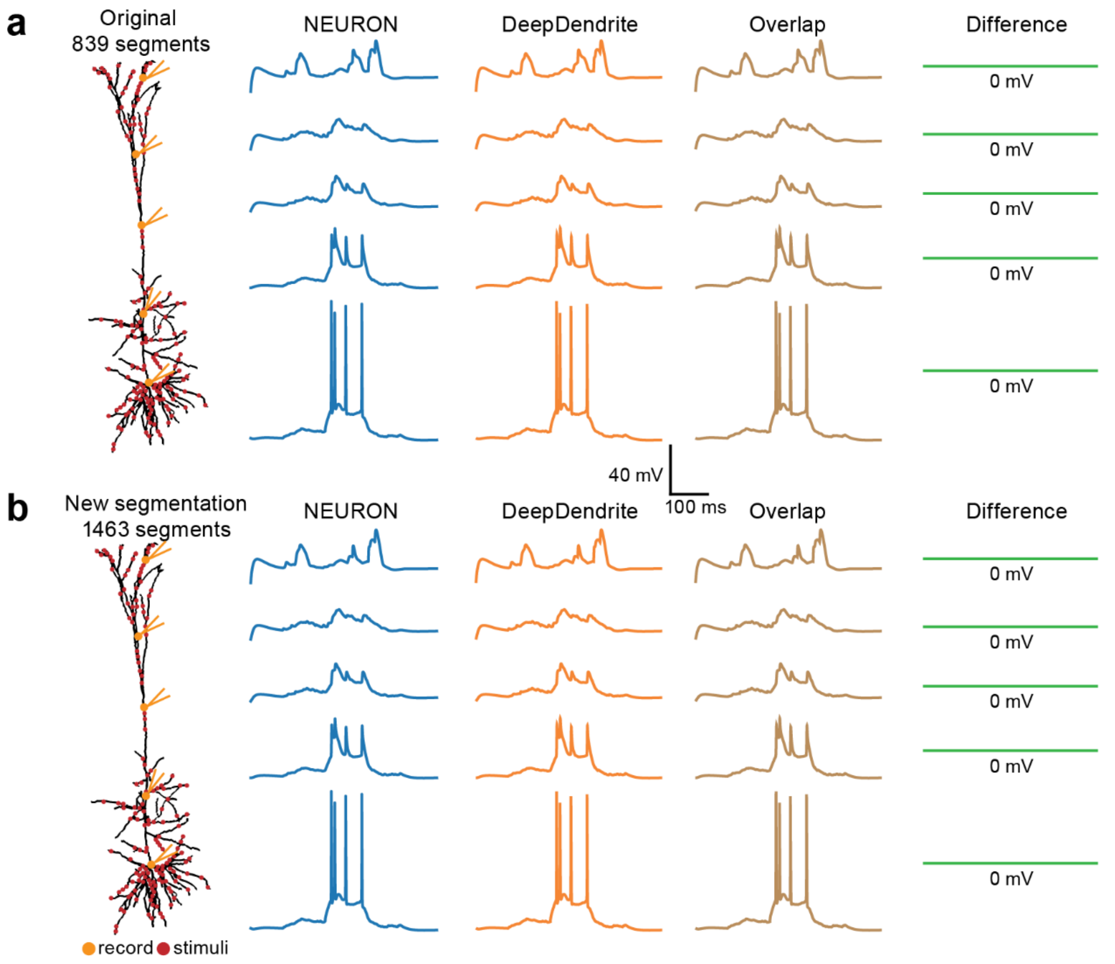

Supplementary Figure 7 | Numerical accuracy of DeepDendrite compared with NEURON under different segmentations and with active dendrties. The layer 5 pyramidal neuron model with active dendrites was taken from *Shai, et al*., 2015. To activate synaptic currents, we introduced 400 Poisson trains operating at a frequency of 2.5 Hz between 80 ms and 400 ms, which encompassed both AMPA and NMDA currents. We also applied a 0.5 nA current to the soma, which persisted for 100 ms . Note the dendritic and somatic responses, including dendritic spikes and somatic bursting, generated by DeepDendrite were identical to those produced by NEURON. **a** Comparison of five voltage traces recorded at the soma and four different dendritic sites simulated using DeepDendrite and NEURON under the original segmentation. **b** Comparison of five voltage traces recorded at the soma and four different dendritic sites simulated using DeepDendrite and NEURON under the new, more fine-grained segmentation.

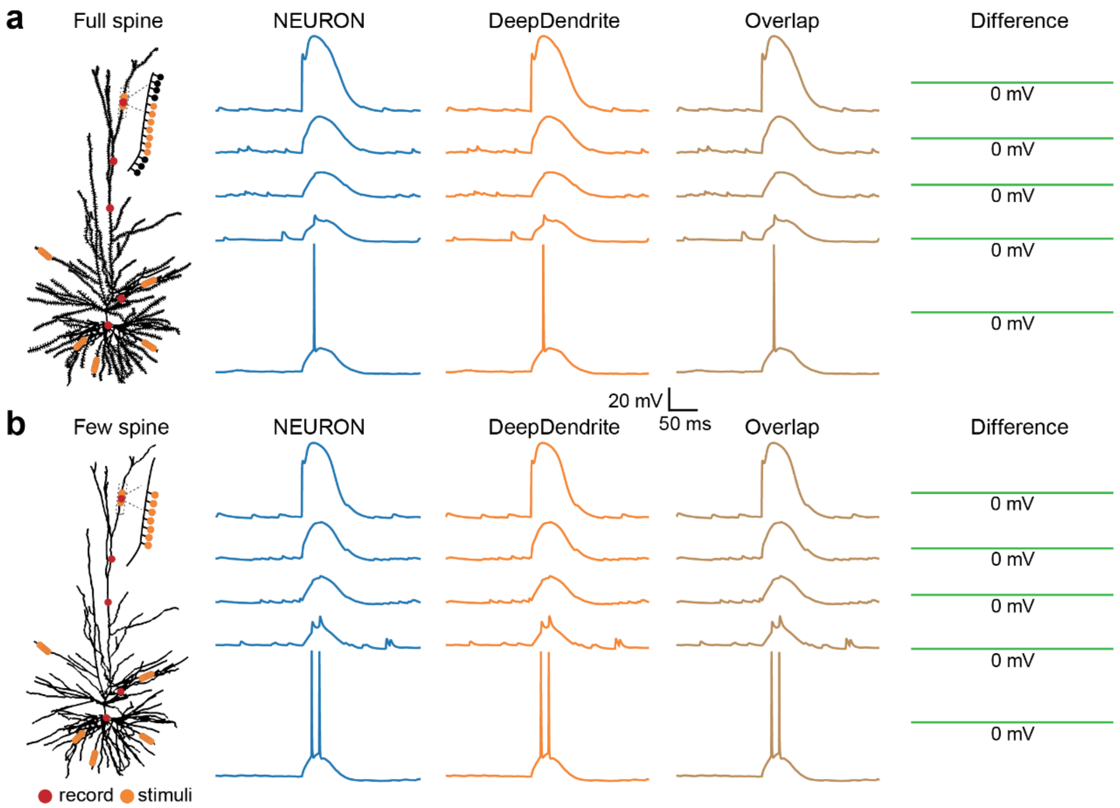

Supplementary Figure 8 | Numerical accuracy of DeepDendrite compared with NEURON under different spine densities. The dendritic and somatic responses generated by DeepDendrite were identical to those produced by NEURON. **a** Comparison of five voltage traces recorded at the soma and four different dendritic sites simulated using DeepDendrite and NEURON on the full-spine model. **b** Comparison of five voltage traces recorded at the soma and four different dendritic sites simulated using DeepDendrite and NEURON on the few-spine model.
